# Substantially Modified Ratios of Effector to Regulatory T Cells During Chemotherapy in Ovarian Cancer Patients Return to Pre-Treatment Levels at Completion: Implications for Immunotherapy

**DOI:** 10.3390/cancers4020581

**Published:** 2012-06-18

**Authors:** Anthony Park, Chindu Govindaraj, Sue D. Xiang, Julene Halo, Michael Quinn, Karen Scalzo-Inguanti, Magdalena Plebanski

**Affiliations:** 1 Department of Immunology, Central Clinical School, Faculty of Medicine, Nursing and Health Sciences, Monash University, Melbourne, Victoria 3004, Australia; E-Mails: Anthony.Park@monash.edu (A.P.); Chindu.Govindaraj@monash.edu (C.G.); Karen.Scalzo@csl.com.au (K.S.-I.); 2 Department of Oncology, Royal Women’s Hospital, Melbourne, Victoria 3052, Australia; E-Mails: Julene.Hallo@thewomens.org.au (J.H.); QuinnM@ramsayhealth.com.au (M.Q.)

**Keywords:** CD4+ T cells, CD8 T cells, regulatory T cells, ovarian cancer

## Abstract

Ovarian cancer is the leading cause of death from gynaecological malignancy. Despite improved detection and treatment options, relapse rates remain high. Combining immunotherapy with the current standard treatments may provide an improved prognosis, however, little is known about how standard chemotherapy affects immune potential (particularly T cells) over time, and hence, when to optimally combine it with immunotherapy (e.g., vaccines). Herein, we assess the frequency and ratio of CD8+ central memory and effector T cells as well as CD4+ effector and regulatory T cells (Tregs) during the first 18 weeks of standard chemotherapy for ovarian cancer patients. In this pilot study, we observed increased levels of recently activated Tregs with tumor migrating ability (CD4+CD25^hi^Foxp3+CD127−CCR4+CD38+ cells) in patients when compared to controls. Although frequency changes of Tregs as well as the ratio of effector T cells to Tregs were observed during treatment, the Tregs consistently returned to pre-chemotherapy levels at the end of treatment. These results indicate T cell subset distributions associated with recurrence may be largely resistant to being “re-set” to healthy control homeostatic levels following standard treatments. However, it may be possible to enhance T effector to Treg ratios transiently during chemotherapy. These results suggest personalized immune monitoring maybe beneficial when combining novel immuno-therapeutics with standard treatment for ovarian cancer patients.

## 1. Introduction

Ovarian cancer affects 200,000 women worldwide each year and is the leading cause of death from gynecological malignancy and the sixth overall cause of death in Australian women [1]. More than three quarters of patients present with advanced stage cancer. Currently, the leading first line treatment includes surgery to debulk the tumour and multiple cycles of carboplatin and paclitaxel chemotherapy [2,3,4]. While this combination treatment is used in developed countries such as Australia, New Zealand, USA and Great Britain, five year survival rates are under 40% [5,6,7] despite initial positive response to therapy in approximately 75% of patients [5,8]. In order to improve treatment efficacy, it may be necessary to consider immunotherapy to augment current first line treatment modalities, however, before this can occur, we must first understand how standard treatments alter lymphocyte homeostasis, in particular the frequencies of various T cell subsets that may respond to immunotherapy.

The frequency of specific T cell subsets has been associated with survival outcomes for cancer patients [9,10,11,12,13,14,15,16,17,18,19,20,21,22]. Elevated levels of regulatory T cells (Tregs), usually characterised as CD4+, CD25+ and/or transcription factor forkhead box P3 (FoxP3) cells, has been widely reported to prevent the immunological clearance of many types of cancer [9]. Tregs are elevated in the peripheral blood of many cancer patients [10,11,12], furthermore, a small sample of breast and pancreas cancer patients shows Tregs in peripheral blood secrete similar suppressive cytokines to those in the tumour and draining lymph node [13]. Under non-pathological conditions Tregs are primarily thought to maintain peripheral tolerance to self-antigens and prevent autoimmune disease [14,15]. Tregs have also been found in both the tumour mass and periphery of ovarian cancer patients [16,17] and increased Tregs or even FoxP3 transcripts within the tumour mass correlate with reduced survival [17,18]. Indeed, multiple studies have shown that regulatory T cells hamper successful anti-cancer vaccine immunotherapy even after tumour removal [19,20,21] and depletion of Tregs in ovarian cancer patients during a recent Phase I/II trial has shown improved anti-tumour immunity [22].

In addition to causing apoptosis of cancer cells, chemotherapy, also affects proliferating peripheral blood lymphocytes [23], and in particular can target for depletion unwanted Tregs [24]. Paclitaxel has been shown to significantly reduce Tregs in both naive and tumour bearing mice [25] and also in patients with non-small cell lung cancer [26]. *In vitro* studies demonstrate that chemotherapeutic drugs not only reduce Treg levels, but also affect their function as incubation of CD4+CD25+ T cells with paclitaxel reduces their FoxP3 expression and suppressive ability [26]. Therefore, while chemotherapy alone may not currently be effective in treating ovarian cancer, these studies have highlighted that understanding the immuno-modulatory effects of the drugs are essential, and chemodrugs can be incorporated into immunotherapeutic schemes (e.g., vaccination) to maximize their effects. However, the optimal timing of such immunotherapy is unknown and warrents further investigation [27], but ideally should be targeted at times of low Treg/suppressor activity and high effector function, and be expected to boost beneficial tumour specific immunity.

Intriguingly, evidence from one recent ovarian cancer study by Wu *et al.* indicates that levels and production of IFN-γ by peripheral blood CD4 and CD8 effector T cells may temporarily increase at the start of chemotherapy while CD4+CD25+ Treg levels temporarily decrease [28], suggesting effector function increases after the first round of chemotherapy. In contrast, Coleman *et al*. have shown that CD8 T cells recover their functionality towards the end of chemotherapeutic treatment and that central (CD45RO+CCR7+) and effector (CD45RO+CCR7−) memory subsets remain stable [29]. Both of these studies used only CD4 and CD25 expression to evaluate Treg levels, and while there is a large degree of controversy as to what truly identifies a Treg, there is good evidence that additional markers such as Foxp3 and CD127 are required as CD4 and CD25 are also expressed on activated/effector CD4 T cell [15]. Precise identification of Tregs will allow a better comparison of their levels to potentially beneficial subsets (for tumour elimination) such as CD4 effector (CD25int) or CD8 central and effector memory T cells, particularly for those that have been activated in the patients (CD38+) [30]. The T cell effector to Treg ratio has been shown to be specifically important in ovarian cancer since a high “CD8+ effector: Treg” ratio within the tumour mass has been shown to promote ovarian cancer patient survival [31]. Such high ratios are also proposed to interfere with potential vaccine efficacy in this patient population. In our study we therefore aimed to carefully determine these ratios in peripheral blood. Since T cells expressing CCR4 can respond to chemoatractants (CCL22) secreted by ovarian cancer cells, and CCR4+ Treg have been associated with enhanced tumour recurrence [32], it may be further timely to address the effect of first line treatment on their frequencies.

Our data shows that even though CD4+ or CD8+ T cell effector to Treg and central memory CD8+ T cell to Treg ratios fluctuate dramatically during the course of chemotherapy, an internal homeostasic mechanism prevails and levels and ratios are restored to pre-treatment values. This largely includes a low T effector/Treg ratio. Using new immunotherapeuties could therefore capitalize on the windows during early treatment, where the T effector/Treg ratios are increased, and focus on ongoing and individualized patient immune status monitoring, to time the administration of vaccines.

## 2. Results

### 2.1. Major Cell Subsets Within the Peripheral Blood of Ovarian Cancer Patients Remain Largely Stable over Standard Chemotherapeutic Treatment

**Figure 1:**
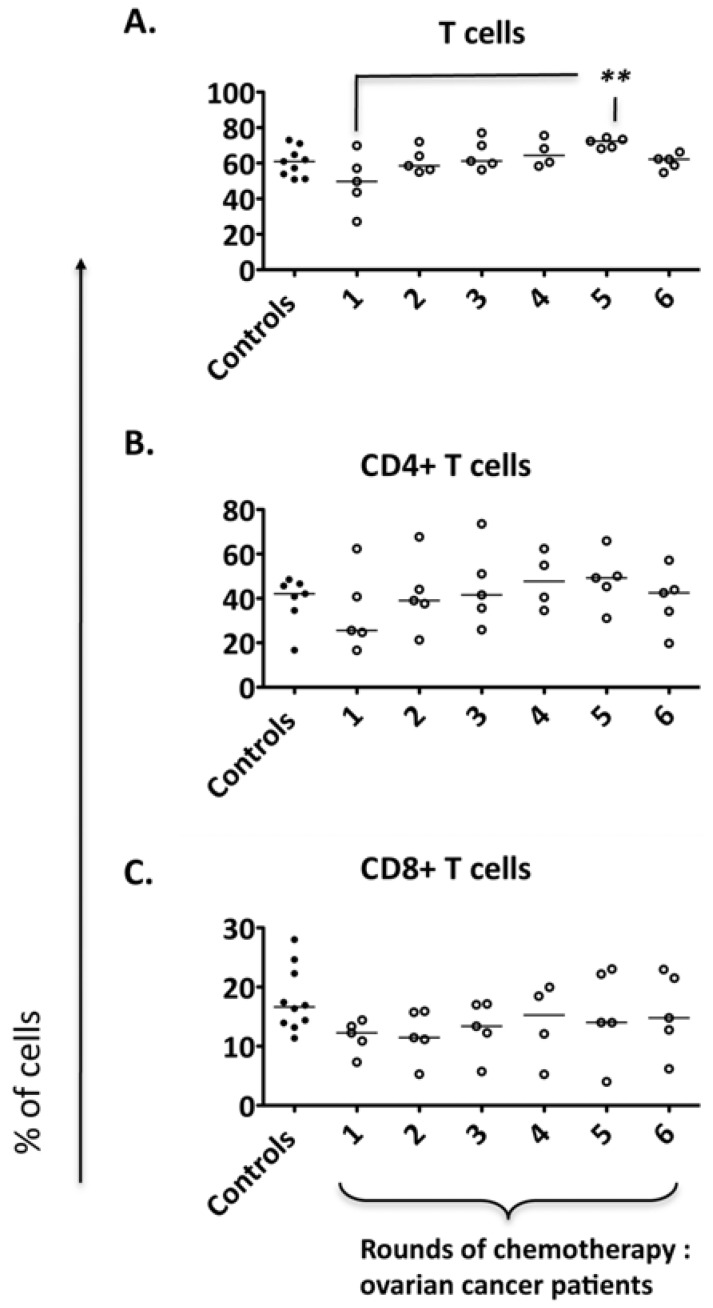
Major subsets within the PBMCs of ovarian cancer patients over the course of standard chemotherapy. Peripheral blood from five ovarian cancer patients was collected over the course of the standard chemotherapeutic treatment. PBMCs of patients and controls (n = 10) were isolated and flow cytometry was performed to determine (**A**) the T cell (CD3+ CD56−) frequency within PBMCs; (**B**) the CD4+ T cell frequency within the T cell fraction (**C**) CD8+ T cell frequency within the T cell fraction. Comparison of the frequency of cell populations between patients and controls: ** *p* < 0.01.

The changes in lymphocyte subset ratios within the peripheral blood of patients with ovarian cancer over the course of first line chemotherapy are unknown. To address this question, we monitored CD4, CD8 and overall T cell frequencies over six rounds of standard chemotherapy with paclitaxel and carboplatin. At the first round of chemotherapy the T cell frequencies for these broad subsets were not significantly different to controls. There was a trend for patients with overall initially low total T cell levels to increase with repeated treatments, with significant increases observed at the timepoint corresponding to 4 rounds of treatment (Figure 1B).

### 2.2. CD8+ T Cell Subsets Fluctuate During the Course of Chemotherapy

**Figure 2:**
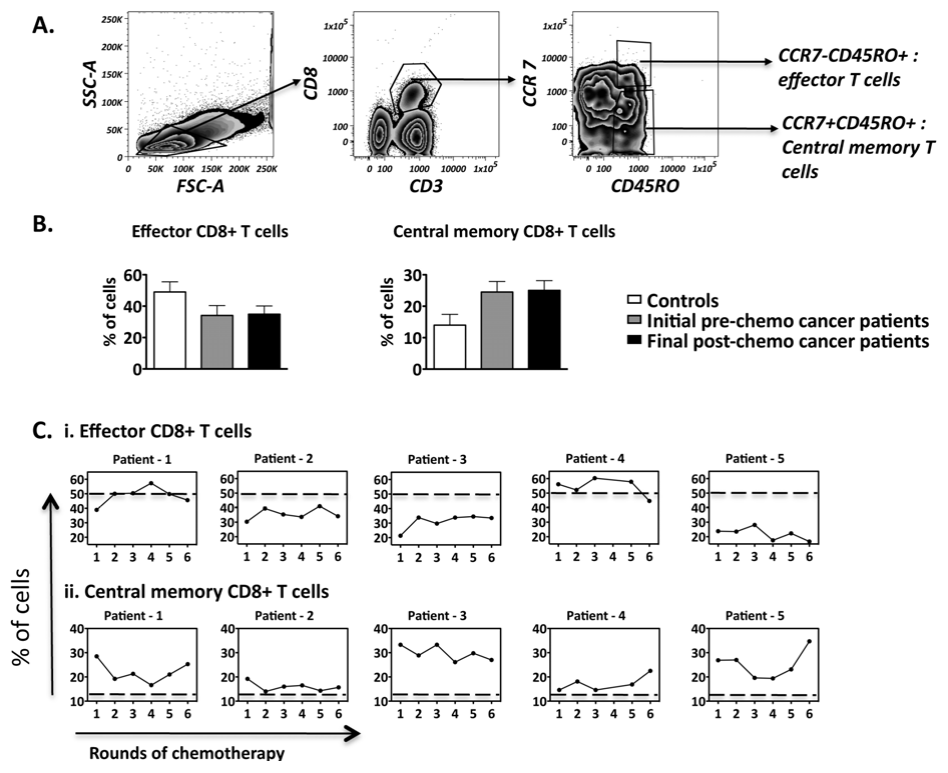
Frequencies of CD8+ T cell subsets present within the peripheral blood of ovarian cancer patients over the course of chemotherapy. Flow cytometry was performed on PBMCS isolated from patients (n = 5) and controls (n = 6). (**A**) The gating strategy employed to identify effector and central memory CD8+ T cells; (**B**) Comparison of the CD8+ T cell subset frequencies between controls and patients; pre-chemo representing 0 rounds while post-chemo represents 6 rounds of chemotherapy; (**C**) Frequency fluctuations of CD8+ T cell subsets of patients over the course of chemotherapy. The dotted line represents the mean frequency of the healthy controls. Comparison of CD8+ T cell subset frequencies between controls and patients: not significant.

It is well established that CD8+ T cells play a major role in the anti-tumor immune response [33,34,35]. Although the overall CD8+ T cell frequencies remained relatively stable, chemotherapy has the potential to selectively deplete proliferating CD8+ T cell subsets, and specifically the central memory T cells that may sustain tumor specific immunity over the long term [36]. To address this possibility in our study, CD8 T cells subsets were further differentiated based on co-expression of CD45RO, which is selectively expressed on activated and memory T cells [30,37,38] and the secondary lymphoid organ-homing surface molecule CCR7 expressed on central memory T cells, yielding effector (CCR7−CD45RO+) and central memory (CCR7+CD45RO+) CD8+ T cells for analysis (Figure 2A).

Our results suggest that most patients have lower CD8+ effector and higher central memory CD8+ T cell frequencies in the periphery when compared to controls (Figure 2B). Importantly, chemotherapy did not alter this trend as the patients retained similar CD8+ T effector and central memory cell levels by their last round of treatment. Interestingly, despite substantial fluctuations over the course of treatment (>40% of initial frequencies), in five out of five patients, the central memory T cells remained higher compared to the healthy controls (average of six healthy controls-represented by dotted lines) by the end of the treatment course (Figure 2C).

**Figure 3:**
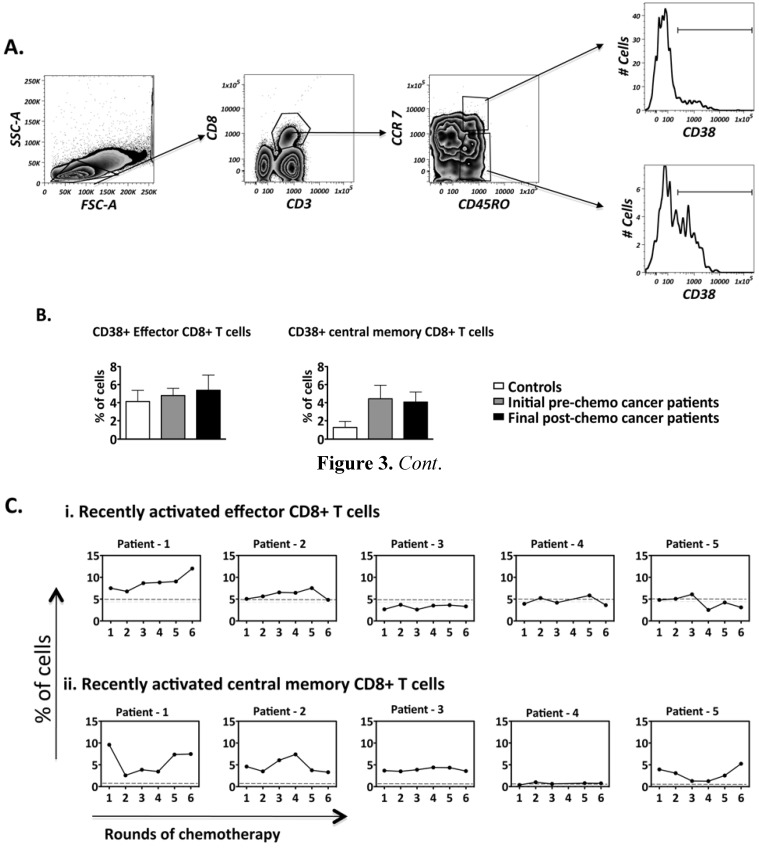
Frequency of recently activated CD8+ T cell subsets present within the PBMCs of patients during the course of chemotherapy. Flow cytometry was performed on PBMCS isolated from controls (n = 6) and patients undergoing chemotherapy (n = 5). (**A**) The gating strategy employed to identify recently activated effector and central memory CD8+ T cells present within the PBMCs; (**B**) Comparison of the CD8+ T cell subsets between controls and patients: pre-chemo representing 0 rounds while post-chemo represents 6 rounds of chemotherapy; (**C**) Frequency fluctuations of CD8+ T cell subsets of patients over the course of chemotherapy. The dotted line represents the mean frequency of the healthy controls. Comparison of CD8+ T cell subset frequencies between controls and patients: not significant.

As no changes in central and effector memory cells were observed over the course of chemotherapy, recently activated memory CD8+ T cells were specifically analysed, as chemotherapy targets proliferating cells [39]. CD38 has been used as a marker for recently activated CD8+ T cells in studies of HIV and viral infections [30,40]. We therefore further analyzed the frequencies of effector and central memory CD8 T cells expressing CD38 (Figure 3A) over the course of chemotherapy. T cells with CCR7−CD45RO+ expression were classified as effector memory cells while cells within CCR7+CD45RO+ expression were classified as central memory T cells [41]. Higher levels of recently activated central memory CD8 T cells were found in patients compared to controls. Again, these levels were largely sustained by of the final chemotherapy treatment time-point, (Figure 3B), despite substantial patient specific fluctuations observed in 3 out of 5 patients (Figure 3C). It was also interesting to note that despite 3 out of 5 patients having lower levels of overall effector T cells (as previously stated in Figure 2C), the levels of recently activated effector CD8+ T cells for all 5 patients were similar to that of healthy controls (Figure 3C). Our data suggests that chemotherapy is not broadly eliminating central memory CD8 T cells, and indeed it shows overall central memory T cells (whether recently activated or not) are found in most patients above the average levels in found in healthy controls.

Pooled data from patients confirmed the same trends, but did not show statistical significance compared to controls, given the wide and non-synchronous fluctuations observed across the patients. Since chemotherapy may only transiently affect the balance of effector to central memory CD8 T cells in each patient, these findings suggest the existence of a strong pre-existing homeostatic mechanism in cancer patients.

### 2.3. Ovarian Cancer Patients Have a Significantly Higher Frequency of Regulatory T Cells and this Frequency Does Not Decrease Despite Chemotherapy

**Figure 4:**
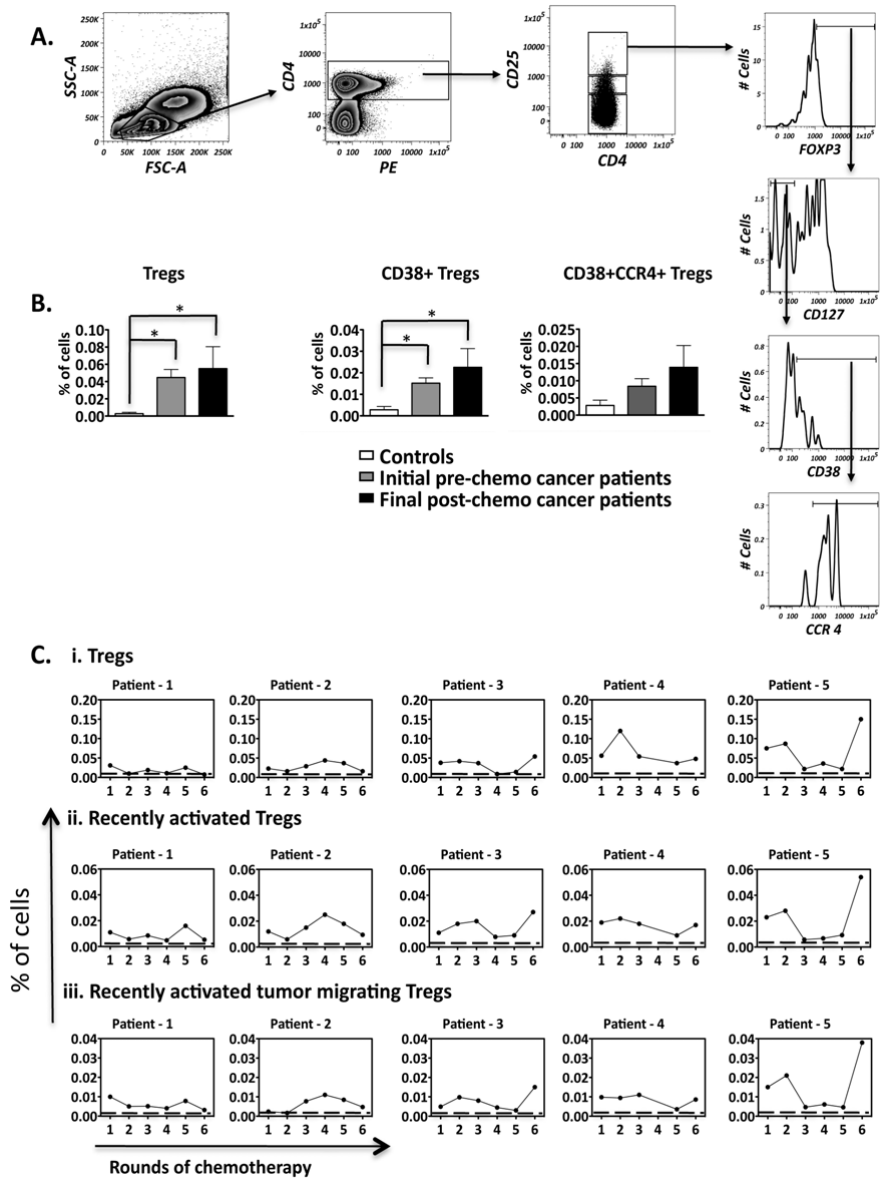
Frequencies of regulatory cell subsets present within the peripheral blood of ovarian cancer patients. (**A**) The gating strategy employed to identify regulatory T cells (CD4+CD25^hi^FOXP3+CD127−) present in the PBMCs; (**B**) Comparison of regulatory T cell subset frequencies between healthy controls (n = 5) and ovarian cancer patients undergoing chemotherapy (n = 5); (**C**) Frequency fluctuations of regulatory T cell subsets of patients over the course of chemotherapy. The dotted line represents the mean frequency of the healthy controls. Comparison of regulatory T cell subset frequencies between controls and patients: * *p* < 0.05.

Regulatory T cells (Treg) are involved in the maintenance of both healthy and pathogenic immune homeostasis in a number of settings, including autoimmunity and cancer [42], and were therefore analyzed for our ovarian cancer patients over the course of chemotherapy. To prevent contamination with activated effector T cells, we used a strict Treg gating strategy, which defined Treg as CD3+CD4+CD25^hi^Foxp3+CD127^low/−^ (Figure 4A). Figure 4B shows that overall Tregs and recently activated Tregs (CD38+ Tregs) frequencies were significantly higher in cancer patients when compared to healthy controls. Moreover, despite substantial fluctuations (>75%), these frequencies did not decrease after chemotherapeutic treatment.

We further looked at the subset of Tregs within this population with potential capacity migrate into an ovarian tumor microenvironment, as determined by the extracellular expression of the CCL22 chemokine receptor, CCR4 [43].

Although not significant, due to substantial variability between patients in this pilot study, the level of recently activated tumor migrating Tregs was higher in 3/5 cancer patients post-chemotherapy when compared to pre-chemotherapy, and fluctuations during treatment largely followed the overall Treg fluctuation patterns. The above results demonstrate that Tregs are not only high compared to controls, but these high levels are sustained over the course of treatment, thus raising the question of whether effective anti-tumour responses could be mounted.

### 2.4. Recently Activated CD4+ Effector T Cells Are Significantly Higher in Ovarian Cancer Patients than Controls and Chemotherapy Does Not Alter this Trend

**Figure 5:**
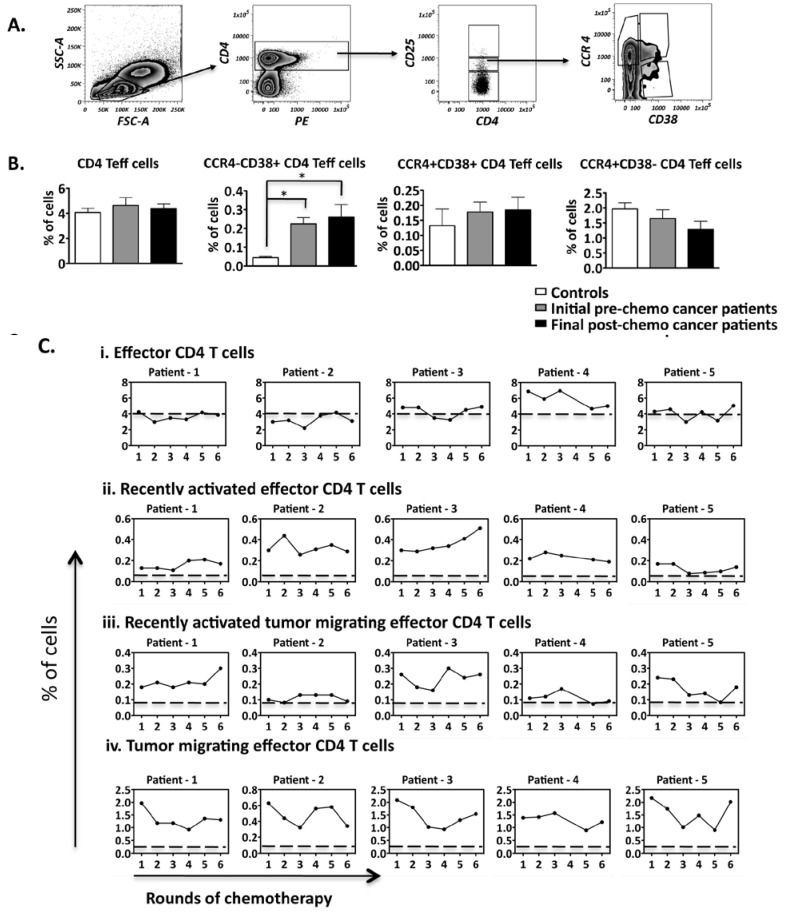
Frequency of CD4+ effector T cell subsets present within the PBMCs of patients. (**A**) The gating strategy employed to identify the different CD4+ T cell subsets present in the PBMCs; (**B**) Comparison of CD4+ T cell subset frequencies between healthy controls (n = 5) and ovarian cancer patients undergoing chemotherapy (n = 5); (**C**) Frequency fluctuations of CD4+ T cell subsets of patients over the course of chemotherapy. The dotted line represents the mean frequency of the healthy controls. Comparison of T cell subset frequencies between controls and patients: * *p* < 0.05.

In order to observe the variation in the frequency of CD4+ effector T cell subsets, we firstly identified CD4+ CD25^int^ T cells as shown in Figure 5A [44]. These CD25^int^ T cells had low levels of FOXP3 expression, further confirming that they are effector T cells (data not shown). We further analyzed expression levels of CD38, which as noted above is a marker for recently activated cells [30,43]. Our results demonstrate that CD38+ CD4+ CD25^int^ effector T cells were significantly higher in patients when compared to controls. Due to the significant change in the CD38+ CD4+ subset in contrast to CD8+ cells, it was further of interest to analyze whether the former would have a tumor migrating capability.

These tumor migrating CCR4+ CD4+ T cell effector subsets, however, were found at similar levels between patients and controls (Figure 5B). Individual data sets represented in Figure 5C further indicate that there are large relative changes in the frequency of CD4+ effector T cell subsets throughput the course of treatment, although the pattern of these fluctuations was patient specific.

### 2.5. Ratios of CD4+ and CD8+ Effector T Cell Subsets to Tregs Fluctuate over the Course of Chemotherapy in a Patient-Specific Manner

**Figure 6:**
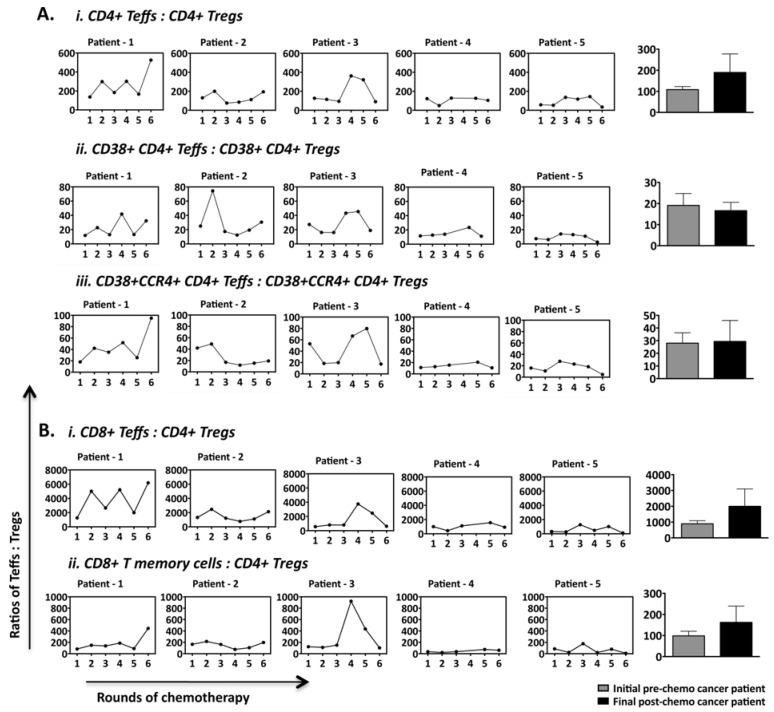
Ratio of CD4+ and CD8+ T cell subsets to Treg subsets identified within PBMCs of cancer patients over standard chemotherapeutic treatment. The ratios of (**A**) CD4+ T cell subsets to their corresponding Treg subsets and (**B**) CD8+ T cell subset to conventional Tregs (CD4+CD25^hi^FOXP3+CD127−) for the individual patients before each round of chemotherapy are represented here. The ratios of all five cancer patients before the first and during the last round of chemotherapy for their corresponding subsets are also shown in concurrent bar graphs (not significant).

Given both effector/central memory CD8+ and CD4+ T cells as well as Tregs fluctuated over the course of chemotherapy, we further analyzed changes in the ratio of these cells. Figure 6 shows that ratios fluctuated dramatically, in a patient specific manner. However, in the majority of patients (4/5), there was a return to the initial pre-chemotherapy ratio during the 18 week of treatment, regardless of how dramatic the extent of the fluctuations during treatment (Figure 6A,B). The above findings reinforce the existence of dominant homeostasis promoting mechanisms that are set during cancer, and are largely resistant to resetting to healthy levels even after tumor elimination and a lengthy course of repeated chemotherapy.

## 3. Discussion

The ratio and nature of T cells is constantly changing in the face of environmental and internal challenges. During cancer, an anti-tumour response is desirable, however, since tumour cells are usually mutated self-cells, a certain level of “control” may be required to avoid potential autoimmune responses. The effect of common cancer treatments on this dynamic process is important to understand in order to improve current therapies and utilize these natural processes more effectively, particularly to be able to combine standard first line treatments with novel vaccine and immunotherapeutic approaches.

Our study focused on investigating the T cell dynamics of patients with ovarian cancer. The overall frequency of total CD4+ and CD8+ T cells remained largely stable over the course of chemotherapy. Although our study did not determine the absolute cell count of these T cells, a previous study found no significant differences in the absolute cell counts of CD4+ and CD8+ T cells between ovarian cancer patients and healthy controls [45]. Effector and central memory CD4+ and CD8+ T cell subset levels, however, were found to fluctuate dramatically, in a patient-specific manner. Intriguingly, despite such individual variations, the final post-chemotherapeutic values were largely restored to initial pre-chemotherapeutic levels in most patients. This uniformity among such variable ovarian cancer patients, including serous, endometrioid, as well as mucinous carcinoma, some of which were metastatic, strongly suggests the presence of a common homeostatic mechanism that governs the regulation of CD4+ and CD8+ T cell subset levels in patients. One of the main regulators of peripheral tolerance are Tregs which are capable of acting on a wide diversity of T cell and non-T cell subsets to maintain homeostasis in the periphery [46,47]. We found Treg frequencies to be higher in cancer patients compared to healthy controls, consistent with several previous reports [12,24]. Importantly, these cells also fluctuated substantially before returning to their initial pre-chemotherapy levels during treatment. Although the frequency of these Tregs were substantially low within CD4+ T cells, the fact that the Treg levels remained similar to the initial pre-chemotherapeutic values for all five patients following surgical debulking and 18 weeks of chemotherapy, suggests that conventionally administered chemotherapy cannot alter the frequency of Tregs. These findings are in contrast to Wu *et al.*, who recently reported that the frequency of Tregs transiently decreased upon administration of the same chemotherapeutic drugs [48]. These conflicting results may be due to the fact that *Wu* and colleagues assessed short-term time points (days 5–28) in contrast to our long-term time point study (week 0–18). Additionally, Wu *et al.* characterized Tregs as CD4+CD25^hi^ T cells, while we used a strict gating strategy (CD4+CD25^hi^FOXP3+CD127^low/−^) to eliminate potential effector T cell contamination. Based on our results, given substantial and patient specific T cell subset fluctuations, we posit that it is necessary to perform individual time-courses to obtain the full picture as to potential changes in T cell subsets, particularly ovarian cancer specific T cells within patient peripheral blood.

We additionally found the ratio of diverse effector and central memory T cell subsets to Tregs similarly returned to pre-chemotherapy levels in patients, despite, in some cases, dramatic fluctuations during treatment. These findings suggest that the homeostatic mechanisms that govern the effector/memory subsets present in the peripheral blood maybe regulated by factors additional to Tregs. A study in breast cancer suggested that effector and regulatory T cells present in the periphery might potentially be regulated by different mechanisms [49]. Future studies to differentiate the mechanisms for different effectors and Treg cell frequency fluctuations may address the possibility that cytokines like IL-2, IL-10 and TGF-β are known to induce Tregs from naive T cells [50,51] and thus, if fluctuating in cancer patients, may promote time-dependently conversion of naive T cells to Tregs rather than effector T cells. Together, the above results suggest that a resilient homeostatic control mechanism gets established in cancer, and re-instates an immune canvas biased more towards immune-suppression in patients compared to controls. However, this conclusion begs a note of caution, since the sampling did not extend beyond the course of chemotherapy. If the chemotherapy substantially reduced the residual tumour burden, it is possible that the subset distributions, after a significant proportion of time may reflect the patterns seen in healthy controls, rather than the observed pre-chemotherapeutic values.

An intriguing result from our study was that post-surgery cancer patients had significantly higher levels of recently activated effector CD4+ T cells as well as Tregs (CD38+) than controls, and they remained significantly increased until the completion of the study (18 weeks). This recent activation of effector T cells suggests that tumor antigens may still persist even after “tumor debulking”, thereby potentially resulting in the generation of anti-tumor effector T cells. However, Tregs are also concurrently activated and may inhibit any existing anti-tumor immunity. Although most of the recently activated Tregs were capable of migrating to the tumor microenvironment (as determined by CCR4+ expression), CD38+ effector T cells were mostly CCR4−. Given that the CD38+CCR4− CD4+ effector T cell subset is likely thus to represent recently activated CD4+ effectors that would not respond to chemoattractants (CCL22) secreted by ovarian tumor cells, this result suggests that in patients, emergent ovarian cancer may promote firstly an influx of CD4 Tregs rather than effector cells. This further highlights the importance of Tregs in generating an immune-suppressive tumor microenvironment for recurrent tumors. It is also notable that despite this being a pilot study involving multiple timepoints from five very different patients receiving the same treatment, there was remarkable consistency in the main findings, both in the overall conclusions and key statistically significant results. Such results give a strong indication that similar studies are worth pursuing in the larger scale and that it may be possible to identify common treatments that may further optimize Treg depletion, while retaining ability of the patients to respond to immunotherapies such as vaccines. We also tested for possible correlations between levels of the different immune T cell subsets with clinical responses, however no clear correlations were obtained, potentially due to the low patient numbers.

Our results therefore suggest adjuvant therapies to eliminate Tregs may need to be used in conjunction with current treatments in order to re-establish effective anti-tumor immunity [52]. Chemotherapeutic drugs alone do not specifically target Tregs, although cyclophosphamide has been found to be an effective Treg depleting agent in cancer patients [24,53,54]. Treg depletion can also be achieved by an IL-2 diphtheria toxin fusion protein (ONTAK), which preferentially targets the IL-2 α chain receptor, CD25, expressed primarily but not exclusively, on Tregs [55]. Depletion of Tregs may potentially enhance the anti-tumor immune response, and thereby prevent tumor recurrences. Therefore, addition of a drug that selectively reduces Tregs to the current treatment regimes may improve the anti-tumor immunity. A recent trial in advanced ovarian cancer patients, however, showed that cyclophosphamide plus carboplatin had the same overall survival as carboplatin plus paclitaxel treatment [56]. On the other hand metronomic (iterative low dose) cyclophosphamide treatment given to end stage cancer patients has been shown to deplete Tregs and enhance anti-tumor immunity [53,57]. This suggests that cyclophosphamide is capable of selectively depleting proliferating Tregs and administrating of iterative low doses may enhance the chances of capturing these actively proliferating Tregs. Prolonged iterative regimen of chemotherapeutic drugs can, however, result in increased toxicity, especially in advanced cancer patients. Therefore, we propose that T cell subsets including Tregs need to be continuously monitored in patients during the course of chemotherapy as T cell subsets fluctuate in an individual specific manner between patients. By administering Treg-depletion therapies at time-points where activated Treg frequencies are elevated for each individual patient, it may be possible to promote preferential depletion of Tregs, thereby enhancing potential anti-tumor immunity, and optimizing the potential reactivity of that patient to co-administered vaccines. This may also result in longer disease-progression free survival of the ovarian cancer patients.

## 4. Experimental Section

### 4.1. Patient and Control Populations

Five ovarian cancer patients were recruited from the Royal Women’s Hospital (RWH) in Melbourne. The project was approved by the RWH ethics committee (RWH Project 07/17). The experiments were conducted with the understanding and the consent of the human subjects. Ten healthy female control samples were obtained from lab volunteers (age 23–52, mean age 34 ± 8.7 years). Patients with diverse primary ovarian cancer (Table 1) were assessed along with sex-matched healthy controls. The mean age of ovarian cancer patients was 51 years. Patients in this study received Carboplatin (AUC5) with paclitaxel (175 mg/msq) over 3 hours every three weeks. Each three-week period corresponded to one standard chemotherapeutic cycle. General adverse toxicity to chemotherapy included generic effects of cytotoxic agents on bone marrow resulting in anaemia, low white blood cell counts and thrombocytopenia. Paclitaxel results in alopecia while carboplatin, especially when used in combination with taxanes (like paclitaxel) can cause neural toxicity.

**Table 1:** Patient characteristics.

Patient	Age	Metastatic	Grade	Cancer Histology
1	40	Yes	Silverberg III	SC
2	37	No	Silverberg III	Mixed serous (90%) and clear cell (10%)
3	59	No	Grade II	Mucinous
4	42	No	AC: Grade IISC: Grade III	Endometrioid AC (65%) and SC (30%)
5	77	Yes	Grade III	Papillary serous carcinoma in the ovaries, omentum and uterus

The age and disease characteristics of the ovarian cancer patients recruited for this study. Some patients were assessed using a specific ovarian cancer Silverberg grading system for prognosis while the rest of the patients were assessed using the standard tumor grading system. Adenocarcinoma: AC; Serous carcinoma: SC.

### 4.2. Antibodies

Mouse anti-CD4 (IgG2a) and rat anti-FoxP3 (IgG2a) were purchased from eBioscience (San Diego, CA, USA). Antibodies to CD3, CD4, CD8, CD56, CD25, CD38, CD45RO, CD127 and CCR4 and were purchased from BD Biosciences (San Jose, CA, USA).

### 4.3. Collection of Peripheral Blood Mononuclear Cells

Approximately 30 mL of peripheral blood was collected in heparinised tubes from each patient at each time point, starting after surgical de-bulking of the ovarian tumour, and subsequently every three weeks just prior to the chemotherapeutic treatment with carboplatin and paclitaxel. Over the course of chemotherapy, a total of six bleeds were collected. Healthy control samples were also collected from 10 volunteers, with 6–10 separate individual samples analysed for each T cell subset analysis as detailed in the Figure legends. Peripheral blood mononuclear cells (PBMC) were separated through Ficoll-Paque (Amersham Pharmacia Biotech, Uppsala, Sweden) density gradients.

### 4.4. Cryopreservation and Recovery of Cryopreserved Cells

In order to reduce variability of analysis for each time-point, all cells were frozen after collection and later thawed on one day for bulk analysis. PBMC (1 × 10^7^/mL) were resuspended in fetal calf serum (FCS) with 10% DMSO and were placed in a pre-cooled isopropanol mediated graduated freezing container and frozen at −70 °C then later transferred into liquid nitrogen. For thawing, vials were warmed in a 37 °C water bath for approximately 30 seconds (or until the ice in the vial had just thawed) and the PBMCs were then washed twice in warm AIM V medium (Invitrogen, Carlsbad, CA, USA).

### 4.5. Flow Cytometry Surface Staining

Cells were resuspended in a blocking solution containing PBS/2% normal human serum (NHS) (Sigma, St. Louis, MO, USA) at a concentration of 5 × 10^6^ cells/mL, and plated out into a 96 well V-bottom plate (Sarstedt, Nümbrecht, Germany) at 100 µL/well. The plate was incubated for a minimum of 30 minutes on ice followed by centrifugation at 1,400 rpm for 4 minutes at 4 °C. After removing the blocking solution, cells were resuspended with antibody cocktails (diluted in blocking solution at appropriate concentrations) and incubated for 30 minutes on ice. Cells were then washed in PBS, and finally resuspended in 100 µL of 1% paraformaldehyde for analysis.

### 4.6. Flow Cytometry Intracellular Staining

Cells (5 × 10^5^) were permeabilised with 100 µL of fixation/permeabilisation buffer (eBioscience) for 30 minutes on ice. Cells were washed once in permeabilisation buffer, and followed by resuspending in 30–50 µL of intracellular antibody solution. Cells were then incubated with antibody for 30 minutes in the dark on ice, then washed twice in 100 µL of permeabilisation buffer and finally resuspended in 100 µL of 1% paraformaldehyde for analysis.

### 4.7. Acquisition and Analysis

AllSamples were read and acquired on a FACS Aria (BD Bioscience). 100,000 to 500,000 events per sample were collected. Analysis was performed using FlowJo software (TreeStar, Ashland, OR, USA).

### 4.8. Statistics Analysis

Statistical analysis was done by a one-way ANOVA between each time-point) and performed on GraphPad Prism 5.0.

## 5. Conclusions

The level of Tregs within ovarian cancer patients is significantly increased when compared to healthy controls. Despite dramatic fluctuations observed in a patient-specific manner during standard chemotherapy, the level of Tregs and the ratio of T effector cells to Tregs return to pre-chemotherapeutic levels. As this was a pilot study involving only five patients, it would be important to replicate these results in a larger patient cohort.
